# 

**Published:** 1889-11-02

**Authors:** 


### NOTICE TO CORRESPONDENTS.

All MS., Letters, Books for Review, and other matters intended for the Editor, should be addressed The Editor, The Lodge, Porchester Square, London, W.

**Notice to Advertisers and Subscribers.**

All Advertisements, Orders for Copies of The Hospital, and Business Communications should be addressed to The Publisher, *not to the Editor*, The Hospital, 139-140, Salisbury-court, Fleet-street, E.C.

**Bound Volumes of "The Hospital."**

Vol. VI., now ready, 4s., post free. Cases for binding, may be had the Publisher, at 1s. 6d. each.

**To Residents, Secretaries, and Matrons**

The Editor will be glad to have the Regulations and each Report as issued by Asylums, Hospitals, Convalescent and Nursing Institutions, as well as those of other Charities, and an early copy in duplicate of all Appeals and Festival Notices, etc., addressed to *The Editor, The Lodge Porchester-square, London, W.* Any superintendent, secretary, matron, or other chief official who regularly sends such information may be registered, on application to the Publisher, to receive free of cost a cover for binding The Hospital in half-yearly volumes.

### Scraps and Gleanings.

Southfleet has sent £17 to Gravesend Hospital.

Richmond will hold Hospital Sunday for the twenty-second time on Nov. 10th.

Sidcup Cottage Hospital Committee already report a building fund of over £1,000.

Kidderminster Infirmary receives £100 from the late Ven. Arch deacon Lea.

The Duke of Westminster has founded, at Chester, a house of shelter for homeless girls.

An electrical machine for the execution of prisoners has been erected in Sing Sing prison, U.S.

Mr. Arnold Caddy, M.R.C.S., has been appointed house surgeon to Leeds General Infirmary.

A ball will be held at the Holborn Town Hall on Dec. 3rd, in aid of the Royal Free Hospital.

A St. Moritz Aid Fund, under Dr. Symes Thompson, helps necessitous invalids to travel to the Engadine.

Cheyne Hospital for Incurable Children issues its fourteenth annual report. Finances are satisfactory.

A congress of Russian medical and scientific men will be held in St. Petersburg from Dec. 27th to Jan. 7th.

Robben Island is to receive a harmonium from an anonymous lady. Books are also being sent to the lepers.

St. Osyth's Home of Rest for Working Girls appeals for funds. Many girls are in want of an autumn holiday.

Miss Conybeare's Cottage Hospital at Axminster only had 42 patients last year. The financial report is satisfactory.

Dr. T. A. Richardson was the only English delegate at the meeting of the Canadian Medical Association in August.

Mrs. Blomfield Moore has given £500 as a thankoffering to the Samaritan Free Hospital for Women and Children, London.

Mr. Henry Rees has been appointed second physician, and Mr. G. F. Aldons second medical officer of Plymouth Public Dispensary.

Dr. Dornil cautions his patients against the use of ice, which he says is often formed of impure water, and transmits the typhoid germ.

Perth has at last seen the necessity for a fever hospital, as the Infirmary is unable at present to take in all the cases of scarlet fever.

His Grace the Duke of Northumberland, K.G., has sent a donation of ten guineas to the London Skin Hospital, 47, Cranbourne-street, W.C.

The Church Society for Providing Homes for Waifs and Strays has received a donation of £300 from the executors of the late Mrs. C. H. L. French.

The Midland Branch of the British Medical Association held a meeting at Melton Mowbray on October 24th. About 15 members were present.

Butchers are benevolent people. After their dinner last week donations amounting to over £2,000 were received for the companies' charities.

Miss Russell succeeds Miss Harris as curator of the museum at the Royal Free Hospital in connection with the London School of Medicine for Women.

Dr. Salome Merritt is delivering lectures on the physiology of the brain and nervous system at the Ladies' Physiological Institute in Boston, U.S.

Three Dublin practitioners are at present ill from fever—viz., Messrs. Horne, Ashe, and Auchinleck. Two of the cases are typhoid and the third typhus.

The Rev. Dr. Hiles Hitchens gave the opening address at the commencement of the winter session of the Zenana Medical College in St. George's-road.

Dr. Felkin contributes to the current number of the *Proceedings* of the Edinburgh Royal Society a paper on "The Geographical Distribution of Disease."

Babies can be bought for £2 in New York. The *World* recently sent out a reporter who found the traffic carried on with less ceremony than the sale of chickens.

Under the happy title of "Medical Amenities," the *Daily News* of Oct. 25th gave an account of an action for damages brought by Mr. F. F. Glanville, surgeon, against Dr. Hennessey.

The Baroness Burdett-Coutts has become a patroness of the Santa Claus Society. Fourteen hospitals and infirmaries were supplied last Christmas with gifts of toys, dolls, etc.

Miss Annie Romberger is said to be the first woman dentist in America. She has a practice of twelve years' standing in Philadelphia out of which she makes an income of £1,200 annually.

Miss Moffat and Miss Bennett, students at the London School of Medicine for Women, have both attained the highest possible marks, and the scholarship of £30 has had to be divided between them.

The late Dr. Ricord was a great punster. Four days before his death a colleague said to him: "Cher maitre, vous n'avez pas mauvaise mine." "I advise you not to take any shares in that *mine*, my boy!" was the reply.

By means of an improved microscope made by Seibert of Wetzlar the internal structure of the anthrax bacillus can be made out. This consists of a series of pearl-like corpuscles, which can be plainly seen to undergo division.

A meeting was held in Manchester lately to consider the desirableness of promoting a memorial to the late Father Damien by helping the establishment of an institute in Belgium for training men for work amongst the lepers of Molokai. A resolution expressing sympathy with the work was carried.

The National Health Society has commenced its winter campaign, and lectures have already been organised, or are being arranged to be held, at Putney, Brixton, Lancaster Gate, St. John's Wood, East Molesey, Chislehurst, Dartford, and other places, on "Sick Nursing," "First Aid," "Domestic Hygiene," and general subjects in connection with health.

